# Regional HLA Differences in Poland and Their Effect on Stem Cell Donor Registry Planning

**DOI:** 10.1371/journal.pone.0073835

**Published:** 2013-09-12

**Authors:** Alexander H. Schmidt, Ute V. Solloch, Julia Pingel, Jürgen Sauter, Irina Böhme, Nezih Cereb, Kinga Dubicka, Stephan Schumacher, Jacek Wachowiak, Gerhard Ehninger

**Affiliations:** 1 DKMS German Bone Marrow Donor Center, Tübingen, Germany; 2 DKMS Life Science Lab, Dresden, Germany; 3 Histogenetics, Ossining, New York, United States of America; 4 DKMS Polska, Warsaw, Poland; 5 Department of Pediatric Hematology and Oncology and Hematopoietic Stem Cell Transplantation, University of Medical Sciences, Poznan, Poland; 6 Internal Medicine I, University Hospital Carl Gustav Carus, Dresden, Germany; Beth Israel Deaconess Medical Center, Harvard Medical School, United States of America

## Abstract

Regional HLA frequency differences are of potential relevance for the optimization of stem cell donor recruitment. We analyzed a very large sample (*n* = 123,749) of registered Polish stem cell donors. Donor figures by 1-digit postal code regions ranged from *n* = 5,243 (region 9) to *n* = 19,661 (region 8). Simulations based on region-specific haplotype frequencies showed that donor recruitment in regions 0, 2, 3 and 4 (mainly located in the south-eastern part of Poland) resulted in an above-average increase of matching probabilities for Polish patients. Regions 1, 7, 8, 9 (mainly located in the northern part of Poland) showed an opposite behavior. However, HLA frequency differences between regions were generally small. A strong indication for regionally focused donor recruitment efforts can, therefore, not be derived from our analyses. Results of haplotype frequency estimations showed sample size effects even for sizes between *n*≈5,000 and *n*≈20,000. This observation deserves further attention as most published haplotype frequency estimations are based on much smaller samples.

## Introduction

With more than 20 million potential stem cell donors in the Bone Marrow Donors Worldwide registry [1], the challenge of “smart” donor recruitment becomes increasingly relevant. Corresponding strategies include recruitment efforts focused on young male donors [2] or on relatives of registered donors with rare human leukocyte antigen (HLA) phenotypes [3], minority donor recruitment programs [4]–[7], and regional priority setting of recruitment activities based on HLA frequency differences [8]–[11].

**Figure 1:**
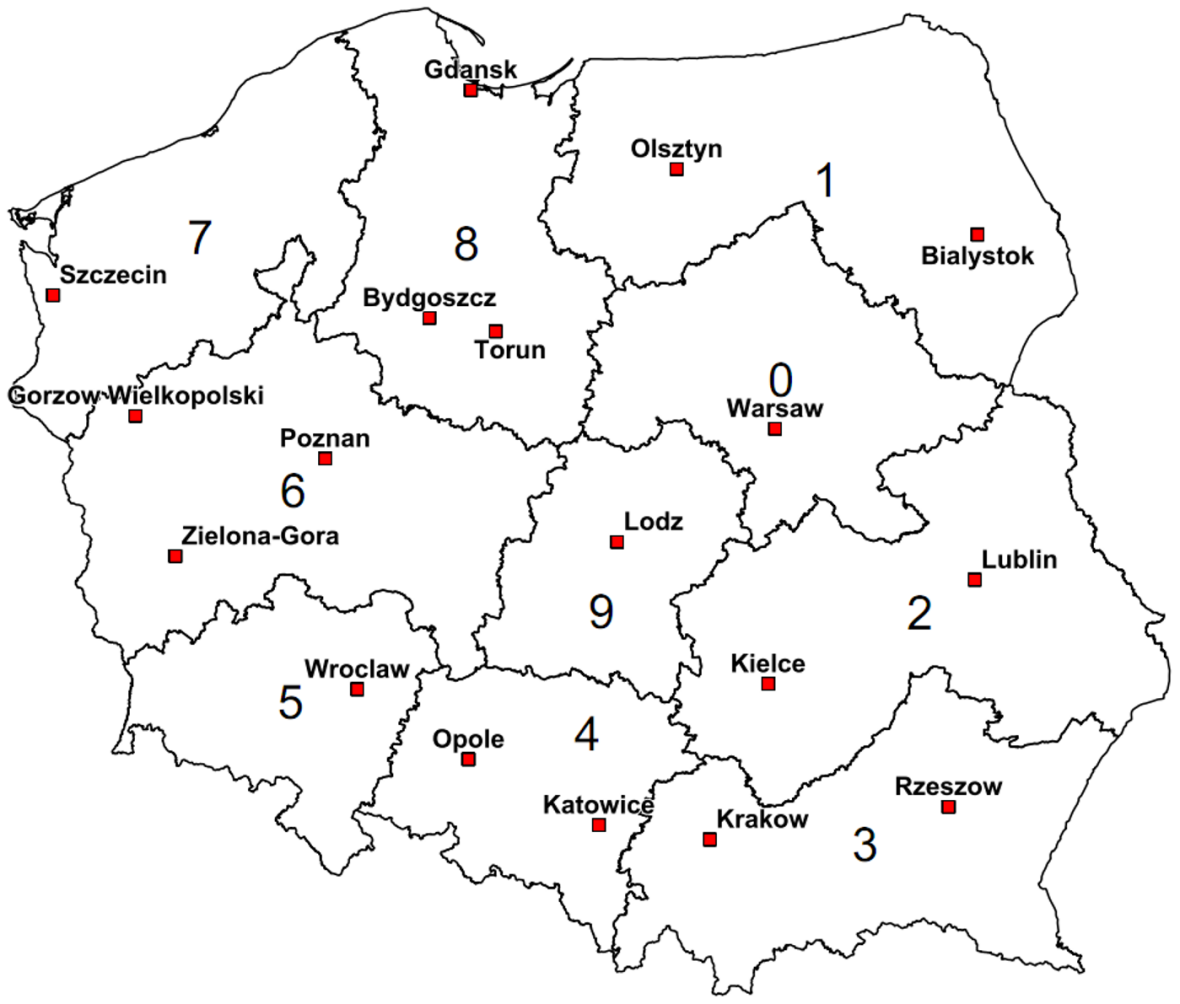
1-digit postal code regions in Poland (© www.bacher.de; © GfK-Geomarketing, Bruchsal, Germany).

Our study was aimed at analyzing regional HLA allele frequency (AF) and high-resolution haplotype frequency (HF) differences in Poland and assessing their implications for an optimized stem cell donor recruitment strategy. Recently, we studied AF and HF of the Polish population based on a large sample (*n* = 20,653) of donors from the DKMS Polska file [12]. However, regional sub-files (based on 10 one-digit postal code regions, Figure 1) with sizes from *n* = 560 (region 9) to *n* = 5,029 (region 4) turned out to be not sufficiently large for a reliable assessment of regional HLA frequency differences. The strong growth of the DKMS Polska donor file since then enabled us to carry out respective analyses on a data set that was approximately six times as large as the original data set.

## Methods

### Data Set

We present analyses based on a raw data set of *n* = 131,473 donors who had been recruited by DKMS Polska from January 2009 to May 2011. 7,724 donors were removed from the raw data set: 6,425 donors were not yet completely HLA-typed (at least for the HLA loci A, B, C, and DRB1 by sequencing-based typing (SBT)) at data retrieval (June 8, 2011), no Polish addresses were available for 894 donors, and 405 donors had unresolved typing ambiguities that resulted most probably from the occurrence of new alleles. The resulting file with *n* = 123,749 donors included the “active” donors (i.e., donors who were still registered and not temporarily unavailable) of the sample with *n* = 20,653 donors that had been studied before. We collected no information about ethnic descent from the recruited donors. A heuristic test had shown no evidence for a substantial admixture of non-Polish donors to the previous data set [12]. Since then, there had been no substantial changes in recruitment practice, thus suggesting that this result was valid also for the larger file analyzed in this study. Besides, we checked the total donor file and the 10 regional sub-files for heterozygosity reduction on antigen level and found no distinct evidence for the Wahlund effect (heterozygosity reduction as result of sub-population structure). The largest reduction could be identified for the HLA-A locus in region 9 with expected and observed heterozygosities of 0.852 and 0.843, respectively. Complete results are given in Supporting Information S1. Taken together, we conclude there is no substantial admixture of non-Polish donors to the data set analyzed in this study.

Regional sub-file sizes ranged from 5,243 (region 9, 0.20% of the respective population) to 19,661 (region 8, 0.47% of the respective population, Table 1). HLA typing was performed at the American Society for Histocompatibility and Immunogenetics (ASHI)-accredited laboratory of Histogenetics (Ossining, NY, USA) and at the ASHI- and European Federation for Immunogenetics (EFI)-accredited DKMS Life Science Lab (Dresden, Germany). HLA typing and data processing were performed as described before [12], [13]. Especially, we combined alleles that differed only by mutations outside the relevant exons or by synonymous mutations using “g” codes [13], [14]. HLA typing results included unresolved ambiguities.

**Table 1:** Regional distribution of study donors (*n* = 123,749) and population figures of Polish 1-digit postal code regions as of June 30, 2010.

1-digit postal code region	Number of donors	Population	Fraction of population (%)
0 (including Warsaw)	18,457	4,485,001	0.41
1 (including Olsztyn)	7,560	2,464,869	0.31
2 (including Lublin)	9,125	4,075,396	0.22
3 (including Krakow)	15,852	5,558,182	0.29
4 (including Katowice)	19,030	5,517,253	0.34
5 (including Wroclaw)	9,182	2,790,326	0.33
6 (including Poznan)	11,521	4,424,734	0.26
7 (including Szczecin)	8,118	2,064,237	0.39
8 (including Gdansk)	19,661	4,173,213	0.47
9 (including Lodz)	5,243	2,633,649	0.20

© GfK-Geomarketing, Bruchsal, Germany.

### Haplotype and Allele Frequencies

In order to assess intra-region diversity, we calculated region-specific HF using an implementation [12] of the expectation-maximization (EM) algorithm [15] that allowed the processing of data with unresolved ambiguities. AF were derived from estimated HF.

Low cumulated HF were regarded as indicator for high intra-region diversity, thus suggesting regions especially suited for donor recruitment efforts. To check for the effects of sub-file sizes, corresponding analyses were also carried out based on the previous data set including *n* = 20,653 donors and on random samples of the regional sub-files with fixed sample size (*n* = 5,000 for each region). Correlations between sub-file sizes and cumulated HF were analyzed using Spearman’s rank correlation coefficient *ρ*. Significance of the *ρ* values was tested by applying a permutation test. Significant correlations between regional sub-file sizes and cumulated HF would indicate that sub-file sizes were too small to allow valid conclusions regarding differences in intra-region diversity.

Three random samples (A, B and C; *n* = 5,000) were generated for each of the regions 4 (large regional sub-file), 6 (medium-scale regional sub-file) and 9 (small regional sub-file) in order to assess the error resulting from drawing samples of equal size (*n* = 5,000) from regional sub-files. For regions 4, 6 and 9, each HF was obtained as mean of three values derived from the respective samples A–C. For determination of the cumulated HF curves, HF of each sample were first cumulated. Then, the mean values were calculated. Similarly, we first calculated sample-specific AF from the corresponding HF and then obtained the AF of regions 4, 6 and 9 as mean values of three sample-specific AF.

We also drew 100 random samples with *n* = 5,000 donors from the total file (*n* = 123,749) and calculated the respective HF distributions. They were compared to respective distributions in the 10 regions (based on samples with size *n* = 5,000) and in a German sample of the same size (taken from a larger sample analyzed before [13]). For each of the regions 4, 6 and 9, mean values of three samples with size *n* = 5,000 were included in this analysis.

Both the estimation of HF from HLA phenotype data using the EM algorithm and the simulation of matching probabilities (MP) (see the corresponding paragraph in this section) require the analyzed populations to be in Hardy-Weinberg equilibrium (HWE). Testing for deviation from HWE using Arlequin [16] was carried out on antigen level as described before [12]. In order to obtain better comparability of the test results for the various regions, we used the random samples (*n* = 5,000) for HWE testing. For each of the regions 4, 6 and 9, the respective A sample was chosen. The *p* values presented in the *Results* section are mean values of three calculations. *p* values were regarded as significant if they were smaller than α = 0.05/40 as 40 combinations of loci and regions were tested (Bonferroni correction).

### Genetic Distances

Genetic distances (GD) between populations were defined as four-locus Euclidean distances of single-locus Cavalli-Sforza and Edwards chord distances [17]. Decisions regarding regional priority setting of recruitment efforts in Poland should take into account the large donor file in Germany as both populations are genetically closely related [6], [12], [18]. We therefore calculated GD between the populations of the various Polish 1-digit postal code regions and the German population [13]. Large distances to the German population would suggest a need for further recruitment efforts in the respective regions. GD between various Polish 1-digit postal code regions were calculated as regions with low distances to many other Polish regions should be favorable for donor recruitment efforts. Donors from such regions should often be full HLA matches to Polish patients.

GD were calculated from random samples with size n = 5,000 for all 10 Polish regions and the German data set [13]. For regions 4, 6 and 9, AF mean values were used as input data.

### Matching Probabilities

We obtained MP by generation of virtual donor and patient files as described before [12]. As starting point for the simulations, we created a virtual donor file with *n* = 1,377,330 donors from the 10 Polish 1-digit postal code regions and from Germany. The file included *n* = 123,749 Polish donors according to Table 1 (column 2) and *n* = 1,253,581 German donors. The number of virtual German donors corresponded with the number of completely typed donors in the donor file of DKMS Germany (date of data retrieval: July 20, 2011). We restricted the number of virtual German donors to the number of real donors with complete HLA typing (high-resolution typing for at least the HLA loci A, B, C and DRB1, not necessarily without unresolved ambiguities) as the use of incompletely typed donors within real donor searches is limited [19]. Virtual donors were created according to the specific HF of their regions or country, respectively, under the assumption of random mating. It was not possible to include the HLA phenotypes of real donors directly into the simulation as they partly included unresolved ambiguities.

We then analyzed the MP increase for Polish patients by recruitment of additional virtual donors from the various Polish 1-digit postal code regions and from Germany. For that purpose, we generated a virtual patient population including *n* = 10,001 Polish patients. The composition of the patient population corresponded to the regional distribution of the Polish population (Table 1, column 3). Patient numbers by region ranged from *n* = 541 for region 7 to *n* = 1,456 for region 3.

Simulations were carried out using HF estimated from the regional random samples of size *n* = 5,000 as the HF estimated from the complete regional sub-files seemed to be potentially biased by sample sizes (see *Results* section). For consistency reasons, German HF were also calculated based on a sub-sample of size *n* = 5000 of the original sample (*n* = 8,862) [13]. Presented results are mean values of three simulation runs A–C. Simulation run A were based on HF derived from the A samples of regions 4, 6 and 9, runs B and C were handled accordingly.

## Results

### Haplotype Frequencies

**Figure 2:**
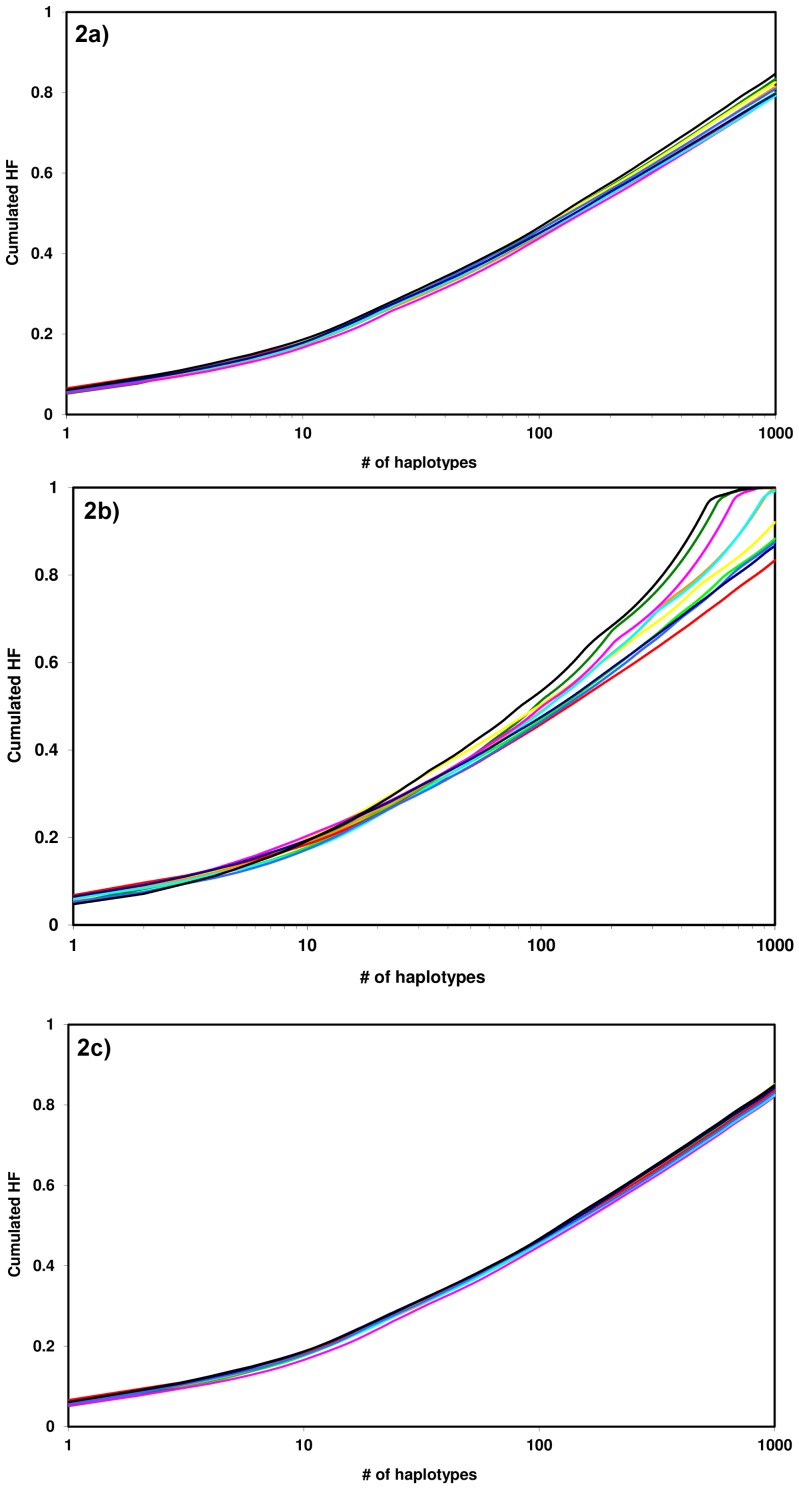
Cumulated HF by Polish 1-digit postal code regions. Light green, region 0; dark green, 1; yellow, 2; orange, 3; red, 4; violet, 5; turquoise, 6; light blue, 7; dark blue, 8; black, 9. HF were estimated from the regional sub-files of the data set including *n* = 123,749 donors (see Table 1 for sub-file sizes, Figure 2a), from the regional sub-files of the data set including *n* = 20,653 donors ([12], Figure 2b), and from regional random samples (see Methods section, Figure 2c). For regions 4, 6 and 9, the points of the cumulated HF curves were calculated as mean values of the respective samples A–C.

Figure 2 shows cumulated high-resolution HLA-A, -B, -C, -DRB1 HF by 1-digit postal code regions. The curves were calculated from the complete data set (*n* = 123,749, Figure 2a), the data set analyzed in [12] (*n* = 20,653, Figure 2b), and the random samples defined in the Methods section (*n* = 50,000, Figure 2c).

Cumulated frequencies of the 10 most frequent haplotypes ranged from 0.166 (region 5) to 0.186 (region 9) for the complete data set (*n* = 123,749). The corresponding intervals for the 100 and 1000 most frequent haplotypes ranged from 0.438 (region 5) to 0.466 (region 9) and from 0.792 (region 6) to 0.847 (region 9), respectively. Statistical testing using Spearman’s rank correlation coefficient *ρ* suggested a negative correlation between sub-file sizes and cumulated HF, especially for large numbers of considered haplotypes. However, the results were not significant at α = 0.05 (Table 2).

**Table 2:** Spearman’s rank correlation coefficients *ρ* between regional sub-files sizes and cumulated HF and corresponding *p* values.

	*n* = 123,749		*n* = 20,653		*n* = 50,000	
# of haplotypes	Spearman’s *ρ*	*p*	Spearman’s *ρ*	*P*	Spearman’s *ρ*	*p*
10	−0.406	0.233	−0.261	0.448	0.006	0.973
100	−0.576	0.081	−0.879	0.001	−0.406	0.233
1000	−0.636	0.052	−0.967	<10^−4^	−0.188	0.595

For *n* = 50,000 (corresponding to ten regional sub-files with size *n* = 5,000), correlations between the regional sub-file sizes of the total data set (*n* = 123,749) and the cumulated HF of the regional random samples are displayed.

HF = haplotype frequency.

Figure 2b shows irregular curves for the smaller sub-files of the previous data set (*n* = 20,653) [12]. Concerned regions included region 9 (*n* = 560), region 1 (*n* = 633), and region 5 (*n* = 715). Cumulated HF were higher for this data set than for the complete data set: between 0.172 and 0.204 for the 10 most frequent haplotypes, between 0.459 and 0.535 for the 100 most frequent haplotypes, and between 0.834 and 1 for the 1000 most frequent haplotypes. Spearman’s *ρ* showed significant negative correlations between sub-file sizes and cumulated HF for 100 and 1000 considered haplotypes at α = 0.05 (Table 2). These correlations were significant also after Bonferroni correction for multiple testing. As a consequence, cumulated HF that were calculated from regional sub-files of the original data set (*n* = 20,653) were regarded as not suited to provide evidence regarding intra-region diversity.

Cumulated frequencies of the 10 most frequent haplotypes ranged from 0.181 to 0.186 (mean: 0.184) for random samples A-C of region 4, from 0.175 to 0.182 (mean: 0.178) for the samples of region 6, and from 0.186 to 0.189 (mean: 0.187) for the samples of region 9. Corresponding ranges for the 100 and 1000 most frequent haplotypes were: 0.460–0.465 (100 haplotypes, region 4, mean: 0.463), 0.450–0.457 (100 haplotypes, region 6, mean: 0.455), 0.467–0.468 (100 haplotypes, region 9, mean: 0.467), 0.835–0.838 (1000 haplotypes, region 4, mean: 0.836), 0.822–0.828 (1000 haplotypes, region 6, mean: 0.825), and 0.848–0.851(1000 haplotypes, region 9, mean: 0.849). We concluded from these inter-sample discrepancies in connection with the inter-region discrepancies (see below) that the sampling error was sufficiently small to allow for further analyses based on random samples.

Cumulated frequencies of the 10 most frequent haplotypes ranged from 0.166 (region 5) to 0.187 (region 9) when regional sub-files of size *n* = 5,000 and the respective mean values of three samples for regions 4, 6 and 9 were considered (Figure 2c). For the 100 and 1000 most frequent haplotypes, the cumulated HF ranged from 0.448 (region 5) to 0.467 (region 9) and from 0.822 (region 5) to 0.851 (region 2), respectively. Cumulated HF for all regions are displayed in Table 3. As expected, Spearman’s *ρ* between the regional sub-file sizes given in Table 1 and the cumulated HF based on random samples of equal size were in good accordance with the null hypothesis (no correlation; Table 2).

**Table 3:** Overview on various parameters related to the suitability of Polish 1-digit postal code regions for further stem cell donor recruitment efforts.

	Cumulated HF						Genetic distances				Matching probabilities											
	10 HT		100 HT		1000 HT		Polish-German		Polish-Polish		*n* = 1,400,000		*n* = 1,500,000		*n* = 1,600,000		*n* = 1,700,000		*n* = 2,000,000		*n* = 2,500,000	
Region	*f*	R	*f*	R	*f*	R	*d*	R	*d*	R	*p*	R	*P*	R	*p*	R	*p*	R	*p*	R	*p*	R
0	0.176	2	0.458	4	0.838	5	0.239	2	0.100	4	0.577	1	0.600	2	0.615	2	0.626	2	0.649	3	0.670	3
1	0.180	5	0.465	9	0.846	7	0.232	5	0.112	9	0.576	7	0.597	8	0.609	10	0.619	10	0.639	10	0.658	10
2	0.181	7	0.463	8	0.851	10	0.246	1	0.101	7	0.577	3	0.599	4	0.614	3	0.625	3	0.647	4	0.669	4
3	0.181	6	0.455	3	0.850	9	0.233	3	0.115	10	0.577	2	0.601	1	0.616	1	0.628	1	0.652	1	0.674	1
4	0.184	8	0.463	6	0.836	4	0.220	9	0.099	2	0.577	4	0.599	3	0.613	5	0.625	4	0.651	2	0.673	2
5	0.166	1	0.448	1	0.822	1	0.228	6	0.099	3	0.576	8	0.598	6	0.611	6	0.622	6	0.645	6	0.666	6
6	0.178	3	0.455	2	0.825	2	0.223	8	0.095	1	0.577	5	0.598	5	0.613	4	0.624	5	0.645	5	0.668	5
7	0.179	4	0.460	5	0.831	3	0.233	4	0.101	6	0.575	10	0.596	9	0.610	9	0.620	9	0.641	8.5	0.661	8
8	0.186	9	0.463	7	0.843	6	0.212	10	0.107	8	0.576	9	0.596	10	0.610	8	0.620	8	0.643	7	0.664	7
9	0.187	10	0.467	10	0.849	8	0.227	7	0.101	5	0.576	6	0.597	7	0.611	7	0.622	7	0.641	8.5	0.661	9

HT = haplotype, *n* = total donor file size (including donors already registered at the starting point of the simulation), *f* = cumulated haplotype frequency, *d* = genetic distance, R = rank. Small ranks suggest high suitability for donor recruitment. Examples: Region 2 has rank 1 concerning Polish-German genetic distances as it has the *highest* distance to the German population. Region 6 has rank 1 concerning Polish-Polish genetic distances as it has the *lowest* mean distance to the other Polish regions.

**Figure 3:**
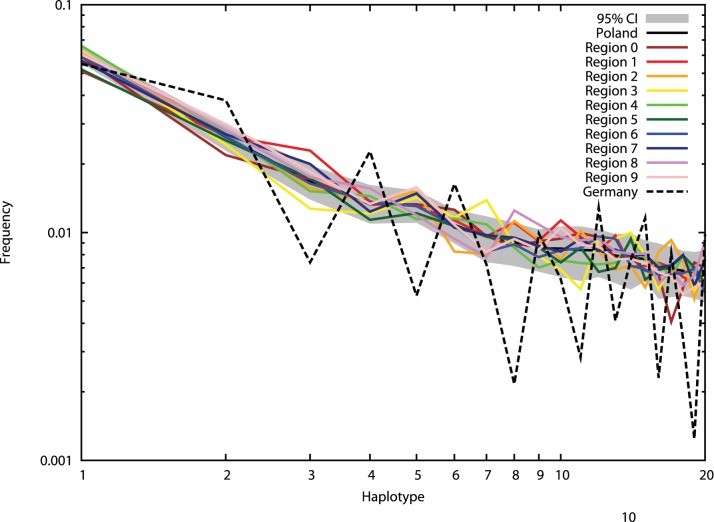
HF of the 20 most frequent haplotypes of the total donor file (*n* = 123,749) in 100 random samples (*n* = 5,000) of this file (displayed are mean values and 95% confidence intervals for each haplotype; mean values and corresponding haplotypes are given in Supporting Information S1), in 10 regional sub-files (*n* = 5,000) and in a German sample (*n* = 5,000).

Figure 3 shows a HF comparison of random samples from the total donor file, regional sub-files and a German sample (n = 5,000 for all samples). The analysis is based on the most frequent haplotypes of the total file. It shows that inter-regional differences within Poland are, with few exceptions, not larger than one would expect from the sampling effect alone.

Complete lists of region-specific AF, HF and cumulated HF are given in Supporting Information S1. For each region, the 10 most frequent haplotypes are displayed in Table 4.

**Table 4:** 10 most frequent HLA-A, -B, -C, -DRB1 haplotpyes for Polish 1-digit postal code regions 0–9.

	Region 0		Region 1		Region 2		Region 3	
Frequency rank	Haplotype	Frequency	Haplotype	Frequency	Haplotype	Frequency	Haplotype	Frequency
1	01∶01g-08∶01g-07∶01g-03∶01	0.0618	01∶01g-08∶01g-07∶01g-03∶01	0.0517	01∶01g-08∶01g-07∶01g-03∶01	0.0603	01∶01g-08∶01g-07∶01g-03∶01	0.0617
2	03∶01g-07∶02g-07∶02g-15∶01	0.0219	03∶01g-07∶02g-07∶02g-15∶01	0.0251	03∶01g-07∶02g-07∶02g-15∶01	0.0293	03∶01g-07∶02g-07∶02g-15∶01	0.0235
3	02∶01g-13∶02g-06∶02g-07∶01	0.0167	02∶01g-13∶02g-06∶02g-07∶01	0.0225	02∶01g-13∶02g-06∶02g-07∶01	0.0164	25∶01g-18∶01g-12∶03g-15∶01	0.0146
4	25∶01g-18∶01g-12∶03g-15∶01	0.0124	25∶01g-18∶01g-12∶03g-15∶01	0.0135	25∶01g-18∶01g-12∶03g-15∶01	0.0147	02∶01g-13∶02g-06∶02g-07∶01	0.0125
5	02∶01g-07∶02g-07∶02g-15∶01	0.0123	02∶01g-07∶02g-07∶02g-15∶01	0.0134	02∶01g-07∶02g-07∶02g-15∶01	0.0124	23∶01g-44∶03-04∶01g-07∶01	0.0119
6	03∶01g-35∶01g-04∶01g-01∶01	0.0112	30∶01g-13∶02g-06∶02g-07∶01	0.0109	02∶01g-27∶02-02∶02g-16∶01	0.0115	02∶01g-07∶02g-07∶02g-15∶01	0.0113
7	23∶01g-44∶03-04∶01g-07∶01	0.0098	03∶01g-35∶01g-04∶01g-01∶01	0.0109	03∶01g-35∶01g-04∶01g-01∶01	0.0094	03∶01g-35∶01g-04∶01g-01∶01	0.0111
8	30∶01g-13∶02g-06∶02g-07∶01	0.0091	24∶02g-13∶02g-06∶02g-07∶01	0.0101	24∶02g-07∶02g-07∶02g-15∶01	0.0094	02∶01g-27∶02-02∶02g-16∶01	0.0091
9	02∶01g-57∶01g-06∶02g-07∶01	0.0087	11∶01g-35∶01g-04∶01g-01∶01	0.0100	02∶01g-57∶01g-06∶02g-07∶01	0.0087	01∶01g-57∶01g-06∶02g-07∶01	0.0085
10	24∶02g-13∶02g-06∶02g-07∶01	0.0085	02∶01g-27∶02-02∶02g-16∶01	0.0092	23∶01g-44∶03-04∶01g-07∶01	0.0086	02∶01g-44∶02g-05∶01g-04∶01	0.0084

	Region 4		Region 5		Region 6		Region 7	
	Haplotype	Frequency	Haplotype	Frequency	Haplotype	Frequency	Haplotype	Frequency
1	01∶01g-08∶01g-07∶01g-03∶01	0.0650	01∶01g-08∶01g-07∶01g-03∶01	0.0535	01∶01g-08∶01g-07∶01g-03∶01	0.0607	01∶01g-08∶01g-07∶01g-03∶01	0.0573
2	03∶01g-07∶02g-07∶02g-15∶01	0.0272	03∶01g-07∶02g-07∶02g-15∶01	0.0251	03∶01g-07∶02g-07∶02g-15∶01	0.0249	03∶01g-07∶02g-07∶02g-15∶01	0.0269
3	02∶01g-13∶02g-06∶02g-07∶01	0.0146	02∶01g-13∶02g-06∶02g-07∶01	0.0164	02∶01g-13∶02g-06∶02g-07∶01	0.0167	02∶01g-13∶02g-06∶02g-07∶01	0.0191
4	02∶01g-07∶02g-07∶02g-15∶01	0.0143	25∶01g-18∶01g-12∶03g-15∶01	0.0125	25∶01g-18∶01g-12∶03g-15∶01	0.0125	25∶01g-18∶01g-12∶03g-15∶01	0.0153
5	03∶01g-35∶01g-04∶01g-01∶01	0.0114	02∶01g-07∶02g-07∶02g-15∶01	0.0122	02∶01g-07∶02g-07∶02g-15∶01	0.0122	02∶01g-07∶02g-07∶02g-15∶01	0.0134
6	25∶01g-18∶01g-12∶03g-15∶01	0.0114	23∶01g-44∶03-04∶01g-07∶01	0.0101	03∶01g-35∶01g-04∶01g-01∶01	0.0105	02∶01g-27∶02-02∶02g-16∶01	0.0099
7	23∶01g-44∶03-04∶01g-07∶01	0.0106	03∶01g-35∶01g-04∶01g-01∶01	0.0098	11∶01g-35∶01g-04∶01g-01∶01	0.0098	30∶01g-13∶02g-06∶02g-07∶01	0.0097
8	01∶01g-57∶01g-06∶02g-07∶01	0.0086	02∶01g-57∶01g-06∶02g-07∶01	0.0092	02∶01g-27∶02-02∶02g-16∶01	0.0095	03∶01g-35∶01g-04∶01g-01∶01	0.0095
9	02∶01g-27∶02-02∶02g-16∶01	0.0079	24∶02g-13∶02g-06∶02g-07∶01	0.0089	23∶01g-44∶03-04∶01g-07∶01	0.0090	24∶02g-13∶02g-06∶02g-07∶01	0.0092
10	02∶01g-44∶02g-05∶01g-04∶01	0.0079	02∶01g-27∶02-02∶02g-16∶01	0.0087	24∶02g-13∶02g-06∶02g-07∶01	0.0087	23∶01g-44∶03-04∶01g-07∶01	0.0090

	Region 8		Region 9					
	Haplotype	Frequency	Haplotype	Frequency				
1	01∶01g-08∶01g-07∶01g-03∶01	0.0608	01∶01g-08∶01g-07∶01g-03∶01	0.0609				
2	03∶01g-07∶02g-07∶02g-15∶01	0.0258	03∶01g-07∶02g-07∶02g-15∶01	0.0299				
3	02∶01g-13∶02g-06∶02g-07∶01	0.0167	02∶01g-13∶02g-06∶02g-07∶01	0.0185				
4	02∶01g-07∶02g-07∶02g-15∶01	0.0144	25∶01g-18∶01g-12∶03g-15∶01	0.0158				
5	25∶01g-18∶01g-12∶03g-15∶01	0.0118	02∶01g-07∶02g-07∶02g-15∶01	0.0134				
6	02∶01g-27∶02-02∶02g-16∶01	0.0108	30∶01g-13∶02g-06∶02g-07∶01	0.0106				
7	03∶01g-35∶01g-04∶01g-01∶01	0.0108	03∶01g-35∶01g-04∶01g-01∶01	0.0105				
8	02∶01g-15∶01g-03∶04g-04∶01	0.0092	02∶01g-27∶02-02∶02g-16∶01	0.0093				
9	24∶02g-13∶02g-06∶02g-07∶01	0.0091	24∶02g-13∶02g-06∶02g-07∶01	0.0088				
10	02∶01g-57∶01g-06∶02g-07∶01	0.0090	02∶01g-15∶01g-03∶04g-04∶01	0.0086				

The HF displayed were estimated from the regional sub-sets of the complete data set (*n* = 123,749).

Testing for deviations from HWE resulted in *p* values <0.05 in 7 of 40 tests: *p* = 0.009 for HLA-B and region 3, *p* = 0.009 for HLA-C and region 3, *p* = 3⋅10^−4^ for HLA-B and region 5, *p* = 0.017 for HLA-B and region 6, *p* = 0.006 for HLA-C and region 6, *p* = 0.048 for HLA-B and region 9, and *p* = 0.009 for HLA-DR and region 9. These *p* values indicated no significant deviation from HWE after Bonferroni correction with the exception of the value for HLA-B in region 5.

### Genetic Distances

The smallest Polish-German GD was observed for region 8 (*d* = 0.2117), the largest one for region 2 (*d* = 0.2457). All respective distances are given in Table 3. The mean Polish-German GD of Polish regions that border to Germany (regions 5–7; 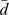 = 0.2279) was only slightly smaller than the corresponding value for regions that do not border to Germany (regions 0–4 and 8–9; 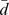 = 0.2299).

Genetic distances between the various Polish regions were considerably smaller than Polish-German distances, ranging from *d* = 0.0872 (between the adjacent regions 4 and 6) to *d* = 0.1354 (between the non-adjacent regions 1 and 3). The largest distance between adjacent regions (*d* = 0.1132) was obtained for regions 2 and 3, the smallest distance between non-adjacent regions was *d* = 0.0894 (regions 0 and 6). The mean distance between the 19 pairs of adjacent regions was 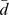 = 0.0991. The corresponding value for the 26 pairs of non-adjacent regions was 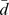 = 0.1060. Region 6 (region 3) had the lowest (highest) mean genetic distance to the other Polish regions (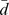 = 0.0954 and 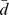 = 0.1148, respectively). The corresponding values for all Polish regions are displayed in Table 3. Visualization of GD by multidimensional scaling using SPSS failed as the resulting stress value was unsatisfactorily high (*S* = 0.309).

### Matching Probabilities

**Figure 4:**
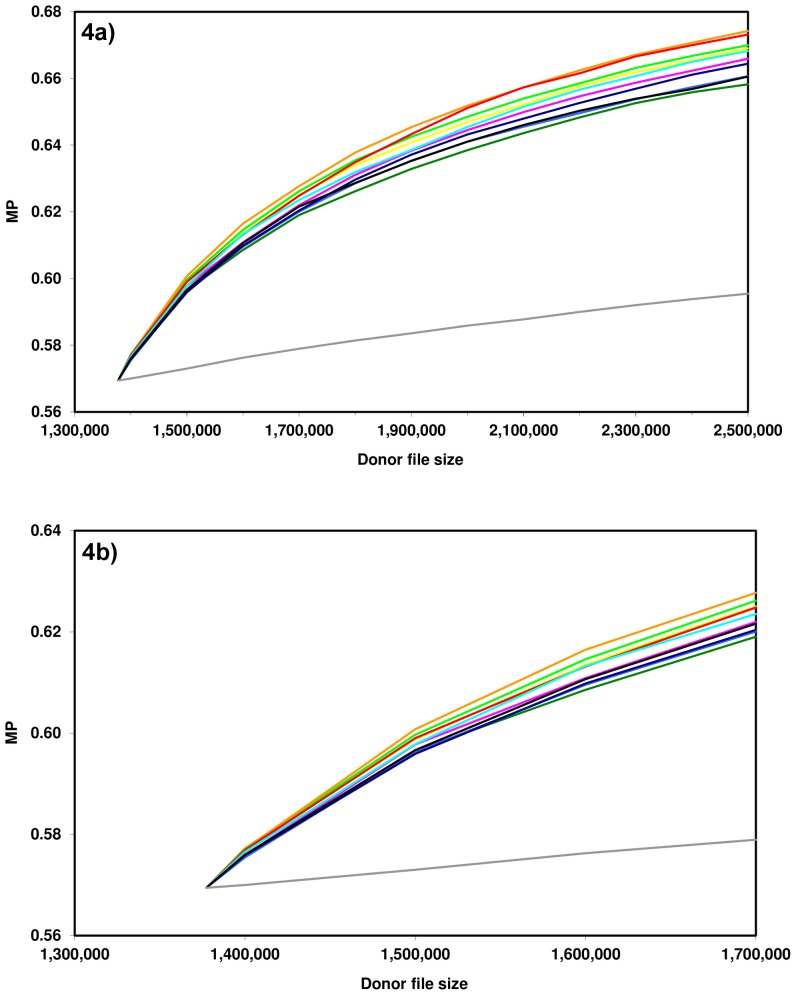
Simulated matching probabilities by donor file size for various populations of newly recruited donors. Light green, Polish 1-digit postal code region 0; dark green, 1; yellow, 2; orange, 3; red, 4; violet, 5; turquoise, 6; light blue, 7; dark blue, 8; black, 9; grey, Germany. Donor file sizes range to *n* = 2,500,000 (Figure 4a) and *n* = 1,700,000 (Figure 4b), respectively.

Figure 4 shows MP for Polish patients by donor file size for various virtual recruitment scenarios. In each scenario, donors from one Polish 1-digit postal code region or from Germany were recruited. At start of the simulation (*n* = 1,377,330), the MP was *p* = 0.569. Simulated MP with new Polish donors ranged from *p* = 0.575 (region 7) to *p* = 0.577 (region 0) for donor file size *n* = 1,400,000, from *p* = 0.596 (region 8) to *p* = 0.601 (region 3) for *n* = 1,500,000, from *p* = 0.609 (region 1) to *p* = 0.616 (region 3) for *n* = 1,600,000, from *p* = 0.619 (region 1) to *p* = 0.628 (region 3) for *n* = 1,700,000, from *p* = 0.635 (region 1) to *p* = 0.652 (region 3) for *n* = 2,000,000, and from *p* = 0.658 (region 1) to *p* = 0.674 (region 3) for *n* = 2,500,000. MP for all regions are given in Table 3.

The MP ranks of the various regions were only slightly influenced by donor file size. Regions 0, 2, 3 and 4 consistently showed relatively high MP while MP for regions 1, 7, 8 and 9 were low for most donor file sizes.

In order to assess determinants of high MP, we analyzed how well the ranks for the parameters given in Table 3 correlated with MP ranks for donor file size n = 1,700,000 by applying Spearman’s rank correlation coefficient. It showed that ranks for GD to the German population correlated most with MP ranks (*ρ* = 0.418), followed by ranks for cumulated frequencies of the 100 most frequent haplotypes (*ρ* = 0.406). Ranks for mean GD to the other Polish regions (*ρ* = 0.127) and cumulated frequencies of the 10 most frequent haplotypes (*ρ* = 0.115) showed only very weak correlations with MP ranks at *n* = 1,700,000. Interestingly, ranks for cumulated frequencies of the 1000 most frequent haplotypes and MP ranks were correlated negatively (*ρ* = −0.248).

The recruitment of additional German donors induced considerably lower MP increases than the recruitment of donors from any of the Polish regions. For *n* = 1,700,000, for example, we obtained an MP of only *p* = 0.579 if German donors were added to the existing file.

## Discussion

We analyzed a very large sample (*n* = 123,749) of registered stem cell donors from Poland with 4-locus high-resolution HLA typing results. The analysis was aimed at identifying geographic regions that are especially suited for further donor recruitment efforts based on the increase of MP for Polish patients.

In Germany, about 5.5% of the population are registered stem cell donors after 20 years of intense donor recruitment efforts. For the most populous Polish 1-digit postal code region (region 3, *n* = 5,558,182, Table 1), this fraction would correspond with approximately 300,000 donors. Therefore, the interval between the starting point of the simulation (*n* = 1,377,330) and approximately *n* = 1,700,000 is most meaningful. Based on the corresponding analyses, there is some evidence that donor recruitment in regions 0, 2, 3 and 4 has an above-average effect on MP while the effect of new donors from regions 1, 7, 8 and 9 on MP seems to be below average. This result suggests that recruitment in the south-eastern part of Poland may improve MP for Polish patients more efficiently than in the northern part of Poland.

Figure 3 shows that HF frequency differences between the various Polish regions are not substantially larger than differences that one would expect from drawing random samples from a nationwide Polish file. As a consequence, the observed recruitment efficiency differences between Polish regions are small. This is especially shown by Figure 4 that allows a comparison of benefits for Polish patients from recruitment of Polish donors in the various regions and of German donors. The figure confirms and refines earlier results [12] that indicate much stronger benefits for Polish patients by recruitment efforts in Poland than in Germany. Intra-Polish efficiency differences seem, compared to the large differences between the Polish regions and Germany, of minor relevance. Therefore, our results do not support a strong regional priority setting of recruitment efforts in Poland.

We had assumed that low cumulated HF, large GD to the German population and small mean GD to other Polish regions were predictors of a strong MP increase by donor recruitment in a specific region. Our results suggest that this assumption may have been too simplistic as only the GD to the German population and the cumulated frequencies of the 100 most frequent haplotypes correlated relevantly with MP.

Based on HF and GD, the MP ranks of some regions as, for example, region 8 are plausible. For other regions, the determinants of the MP ranks remain difficult to understand. Region 3, the region with the highest MP increases, is an example for this group of regions. One may speculate that larger HLA frequency differences between the various regions and/or larger sample sizes would have led to more traceable results.

We analyzed large regional sub-files with sizes from *n* = 5,243 (region 9) to *n* = 19,661 (region 8) donors. Nevertheless, we observed negative correlations between cumulated HF and sub-file sizes (Figure 2a; Table 2, columns 2 and 3) suggesting that high-resolution HF estimations based on large samples with *n*≈5,000–20,000 individuals are biased by donor file sizes. This observation is consistent with results of a comparison of German high-resolution HF estimated from data sets of sizes *n* = 8,862 [13] and *n* = 145,403 [20]. As most published studies regarding population-specific HF distributions are based on much smaller samples [21], there is need for further quantification of the observed bias. At least three errors need to be considered: First, the sampling error increases with decreasing sample size. Second, small sample sizes lead to a systematic underestimation of the diversity of the underlying populations as some haplotypes that are present in the populations are not included in the samples. As a consequence, the frequencies of the remaining haplotypes are generally overestimated. This effect caused the negative correlation between sample sizes and cumulated HF in our study. Third, small sample sizes cause irregular cumulated HF curves that include sharp bends as artifacts (Figure 2b). These sharp bends are caused by several haplotypes that are estimated to occur, for example, once in the sample corresponding with a resulting frequency of 1/(2*n*) with sample size *n*.

Summarized, we analyzed a very large data set (*n* = 123,749) of stem cell donors from all Polish regions. We found evidence that recruitment in regions 0, 2, 3 and 4 increased MP for Polish patients most efficiently. However, differences between the Polish regions were too small to allow a recommendation for strong regional priority setting of donor recruitment efforts. Our calculations using the EM algorithm raised questions regarding the accuracy of HF estimations even for large samples in the range of ≈5,000–20,000 donors. This question deserves further attention as most current studies including HF estimations are based on considerably smaller samples.

## Supporting Information

Supporting Information S1 The supporting information file “Supporting_Information_S1.xls” contains seven Tables. In the first four Tables, the allele frequencies of HLA-A*, HLA-B*, HLA-C*, and HLA-DRB1* alleles are listed. In the fifth and the sixth Table, the haplotype frequencies and the cumulated haplotype frequencies are provided, respectively. The last table lists and compares the expected and observed heterozygosity to illustrate the Wahlund effect.
(XLS)
